# Molecular Characterization of *Cryptosporidium* spp. in Cultivated and Wild Marine Fishes from Western Mediterranean with the First Detection of Zoonotic *Cryptosporidium ubiquitum*

**DOI:** 10.3390/ani12091052

**Published:** 2022-04-19

**Authors:** Samantha Moratal, María Auxiliadora Dea-Ayuela, Alba Martí-Marco, Silvia Puigcercós, Naima María Marco-Hirs, Candela Doménech, Elena Corcuera, Jesús Cardells, Victor Lizana, Jordi López-Ramon

**Affiliations:** 1 Servicio de Análisis, Investigación y Gestión de Animales Silvestres (SAIGAS), Veterinary Faculty, Universidad CEU-Cardenal Herrera, Tirant lo Blanc Street 7, Alfara del Patriarca, 46115 Valencia, Spain; samantha.moratalmartinez@uchceu.es (S.M.); alba.martimarco@uchceu.es (A.M.-M.); puigomsil@gmail.com (S.P.); naimasuntesausias@gmail.com (N.M.M.-H.); zapatacandela@hotmail.com (C.D.); e.corcuera97@gmail.com (E.C.); jcardells@uchceu.es (J.C.); victor.lizana@uchceu.es (V.L.); jordi.lopez1@uchceu.es (J.L.-R.); 2 Pharmacy Department, Universidad CEU-Cardenal Herrera, Santiago Ramón y Cajal Street, Alfara del Patriarca, 46115 Valencia, Spain; 3 Wildlife Ecology & Health Group (WE&H), Veterinary Faculty, Universitat Autònoma de Barcelona (UAB), Travessera dels Turons, Bellaterra, 08193 Barcelona, Spain

**Keywords:** *Cryptosporidium*, genetic characterization, marine fish, Mediterranean, zoonotic

## Abstract

**Simple Summary:**

*Cryptosporidium* is a widespread pathogen that infects a broad range of vertebrates, including humans, in which it is one of the main causes of diarrhea worldwide. Marine fishes also harbor *Cryptosporidium* species, including zoonotic ones. The goal of this study is to evaluate the presence of *Cryptosporidium* species in edible marine fishes using molecular tools. The area of study, located in the Western Mediterranean, is an important area for marine fish production and capture. The following three groups were studied: cultivated fish, wild fish that aggregate in the surroundings of marine fish farms and wild fish from extractive fisheries. Results show that the most affected group is the group of wild fish from the vicinity of fish farms. Two species were mainly identified, *C. molnari* (fish specific) and zoonotic *C. ubiquitum*. The presence of zoonotic *C. ubiquitum* in two European sea bass (*Dicentrarchus labrax*) highlights a potential risk for fish consumers.

**Abstract:**

Fish not only harbor host-specific species/genotypes of *Cryptosporidium*, but also species like zoonotic *C. parvum* or anthroponotic *C. hominis*, which can pose a risk for fish consumers. This study aims to investigate fish cryptosporidiosis in an important aquaculture and fishery area of the Western Mediterranean (Comunidad Valenciana, Spain). We analyzed 404 specimens belonging to the following three groups: cultivated fish (N = 147), wild synanthropic fish (N = 147) and wild fish from extractive fisheries (N = 110). Nested PCR targeting the 18S rRNA gene, followed by sequencing and phylogenetic analysis, were performed. Positive isolates were also amplified at the actin gene locus. An overall prevalence of 4.2% was detected, with the highest prevalence in the synanthropic group (6.1%). *C. molnari* was identified in thirteen specimens from seven different host species. Zoonotic *C. ubiquitum* was detected in two European sea bass (*Dicentrarchus labrax*). One isolate similar to *C. scophthalmi* was detected in a cultivated meagre (*Argyrosomus regius*), and one isolate, highly divergent from all the *Cryptosporidium* species/genotypes described, was identified from a synanthropic round sardinella (*Sardinella aurita*). This study contributes to increasing the molecular data on fish cryptosporidiosis, expanding the range of known hosts for *C. molnari* and identifying, for the first time, zoonotic *C. ubiquitum* in edible marine fishes, pointing out a potential health risk.

## 1. Introduction

Fish consumption has increased at an average annual growth rate of 3.1%, far above the rate of annual world population growth (1.6%). This high demand for fish products (156 million tonnes intended for human consumption in 2018) is supplied by capture fisheries and aquaculture production [1]. In the next decade, a notable increase in production is expected, attributable to the fast growth of aquaculture, whose production will exceed that of capture fisheries [2]. Concerning marine finfish, monitoring of fish stocks indicates a continue decline of marine fishery resources, especially in the Mediterranean and Black Sea, with 62.5% of marine stocks at unsustainable levels [1,3]. In this context, coastal and marine aquaculture has an important role as a sustainable source of fish protein.

*Cryptosporidium* is an important water- and food-borne pathogen all over the world. It is considered the fifth most important food-borne pathogen, with more than 8 million cases of food-borne illness reported annually [4]. It is one of the main causes of diarrhea worldwide, especially in young children [5], and may even be a life threatening pathogen in immunocompromised people [6]. Cryptosporidiosis is not exclusive to humans, as there are several *Cryptosporidium* species infecting a wide range of wild and domestic vertebrates [7,8].

Concerning fishes, *Cryptosporidium* has been detected in several hosts, including cultivated and wild freshwater and marine species, as well as ornamental fishes. *Cryptosporidium* spp. characterization in fish is based mainly on morphological description and, chiefly, on molecular methods, which are essential to identify species, genotypes and subgenotypes [9]. Currently, the following five species are recognized as specific to fish hosts: *C. molnari*, originally characterized in wild gilthead seabream (*Sparus aurata*) and European seabass (*Dicentrarchus labrax*) [10,11]; *C. scophthalmi*, which has been only characterized genetically in wild turbot (*Scophthalmus maximus*) [12]; *C.m huwii* from guppy (*Poecilia reticulata*), golden tiger barb (*Puntigrus tetrazona*) and neon tetra (*Paracheirodon innesi*) [13,14,15]; *C. bollandi* from Oscar fish (*Astronotus ocellatus*) [16]; and recently proposed *C. abrahamseni* n. sp. from red-eye tetras (*Moenkhausia sanctaefilomenae*) [17]. Additionally, several genotypes specific to fish (piscine genotypes) have been described [18].

Non-piscine host-specific species and genotypes have been detected in fishes too. The high environmental oocyst resistance [19] allows the contamination of aquatic environments with oocysts coming from terrestrial species (generally by fecally contaminated wastewater or agricultural run-off), which is accumulated in marine organisms like shellfish and fish [20]. Zoonotic *C. parvum*, anthroponotic *C. hominis*, *C. xiaoi*, *C. scrofarum* and rat genotype III have been detected in fishes [18]. Although it has not been confirmed that these pathogenic species are actually infecting fish hosts, and fish-borne cryptosporidiosis outbreaks have not been reported, their presence in edible fish suggests, at any rate, a potential risk for public health. *Cryptosporidium* has already been detected in fillets [21], suggesting a potential cross-contamination risk during evisceration. Furthermore, there are fish species that are consumed ungutted and, occasionally, raw or undercooked [22].

Comunidad Valenciana is the largest marine aquaculture producer in Spain, generating 16,353 tonnes in 2020 [23]. This activity coexists with high extractive fishing activity, providing an interesting region in which to study fish cryptosporidiosis, both in farmed and wild captured marine fishes. Therefore, the aim of this study is to estimate the prevalence of fish cryptosporidiosis in this region and to identify the *Cryptosporidium* species present, with special focus on zoonotic species.

## 2. Materials and Methods

### 2.1. Study Area and Fish Sampling

The study was performed in Comunidad Valenciana, a region located in Eastern Spain, whose marine area belongs to the Levantine–Balearic Demarcation. Research was conducted in four on-growing off-shore aquaculture farms belonging to the Agrupación de Defensa Sanitaria Acuicultura de la Comunidad Valenciana (ADS ACUIVAL). These farms, located at a mean distance of 1.87 nautical miles from the coast and at an average depth of 35.75 m, are dedicated to the fattening of European sea bass, gilthead seabream and meagre (*Argyrosomus regius*) in floating pens. Cultivated fish are separated from synanthropic fish that aggregate around floating pens only by nets of different mesh sizes, allowing the free circulation of water. Fish sampling was conducted from July 2020 to October 2021, mainly in the autumn and summer seasons. During the second year of the study (March to June 2021), fish from extractive fisheries, mainly trawling, were obtained from four different fish markets in the same marine demarcation. These fish markets were distributed along the coast of Comunidad Valenciana and were chosen to be as far away as possible from the farms, to guarantee that these fishes constituted an independent group.

A total of 404 fishes were sampled, from the following three different groups: (1) cultivated fishes from the four study farms study (N = 147); (2) wild synanthropic fishes caught in the surroundings of the floating pens from these four farms (N = 147); (3) and wild fish from extractive fisheries obtained at the fish markets (N = 110) (Table 1). Fishes from groups 1 and 2 were stunned and slaughtered following the standard procedures used at Mediterranean marine farms (immersion into a slurry ice solution), that were approved by the project financing entity.

Sampled fish were refrigerated and transported to the Parasitology laboratory at CEU Cardenal Herrera University (Alfara del Patriarca, Valencia, Spain), to be processed within the first 24 h after death. Species determination, body weight and total body length were recorded, and dissection was performed for each individual using sterile dissection material. Gastrointestinal tissue scrapings mixed with intestinal contents were preserved at −20 °C until DNA extraction.

### 2.2. DNA Extraction and Molecular Detection of Cryptosporidium spp.

DNA extraction was performed using an NZY Tissue gDNA Isolation Kit (Nzytech genes & enzymes, Lisbon, Portugal), according to the manufacturer’s instructions. Preliminary steps for samples containing stool were also applied, in order to maximize the efficiency of the extraction.

Samples were tested for *Cryptosporidium* spp. following a nested PCR protocol to amplify a ≈ 578 bp fragment of the 18S rRNA gene, as described by Ryan et al. (2003) [24]. All PCR runs included a positive control, consisting of DNA from *C. ubiquitum* isolated from infected farmed lambs, and a negative control without DNA template. Positive isolates were also amplified at the actin locus using *Cryptosporidium* spp. specific actin primers [25] and primers specifically designed for piscine-derived *Cryptosporidium* [26]. Products of the secondary reactions were visualized on 1.5% agarose gel stained with RedSafe TM Nucleic Acid Staining solution (iNtRON Biotechnology, Seongnam, Republic of Korea).

### 2.3. Sequence and Phylogenetic Analysis

Positive samples were Sanger-sequenced by the sequencing service of the Genomics Department at Principe Felipe Research Centre (Valencia, Spain), using an ABI Prism 3730 sequencer (Applied Biosystems, Foster City, CA, USA). The nucleotide sequences obtained were visualized using the Chromas version 2.6.6 (Technelsyum DNA Sequencing Software, South Brisbane, QLD, Australia) and compared with *Cryptosporidium* spp. sequences deposited in the NCBI GenBank database, using the online BLAST tool (http://blast.ncbi.nlm.nih.gov/blast (accessed on 24 February 2022). Phylogenetic analyses were conducted using MEGA version 11 [27]. Sequences were aligned to selected reference sequences, pairwise distance matrixes were calculated, and phylogenetic trees for the 18S rRNA and actin genes loci were constructed by the Maximum Likelihood (ML) method using the Tamura 3-parameter substitution model. Bootstrap tests were conducted on 1000 replicates.

Partial sequences of the 18S rRNA and actin genes from this study were deposited in GenBank under the accession numbers OM574856-OM574862 and OM650810-OM650814.

### 2.4. Data Analysis

Prevalence, expressed as mean ± standard error (SE), was calculated for each group. Fisher’s exact test was applied to assess differences between the groups’ prevalence. The significance level was set at a *p* value ≤ 0.05. Mean weights and mean total body lengths ± standard deviation (STD) were calculated. Analyses were performed on R software [28].

## 3. Results

### 3.1. Prevalence

**Figure 1:**
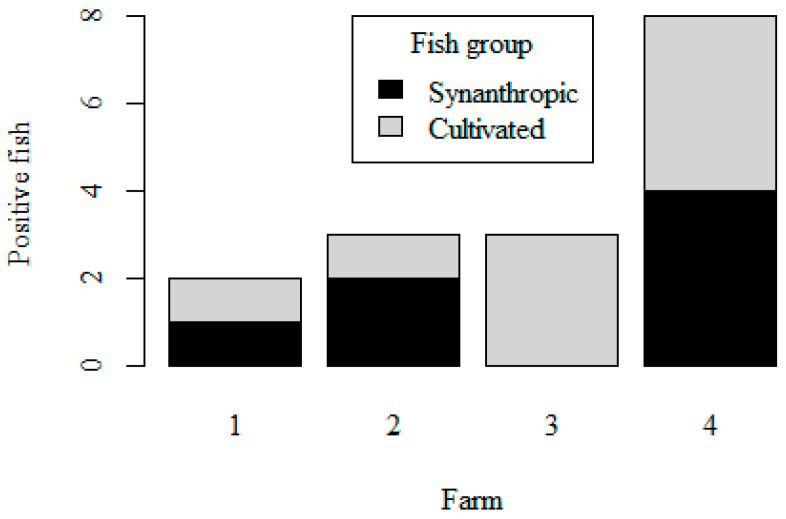
Number of positive fishes according to group and location (only applied to Farms).

*Cryptosporidium* spp. were detected in 17 out of 404 samples (4.2 ± 1%). Prevalence in the cultivated group was 4.8 ± 1.8% (7/147), while prevalence in wild fish was 3.9 ± 1.2% (10/257). Among wild fish, prevalence was higher in the group of synanthropic fishes (6.1 ± 2%; 9/147), with only one fish infected from extractive fisheries (0.9 ± 0.9%; 1/110). The difference in prevalence between these two groups was statistically significant (*p* = 0.047). Positive isolates were found in synanthropic fish from the four marine farms studied and in cultivated fish from three of these farms (Figure 1).

*Cryptosporidium* spp. were detected in all of the three cultivated species analyzed, in four European seabass, two gilthead seabream and one meagre. Regarding synanthropic fishes from farm surroundings, *Cryptosporidium* spp. were detected in the following five species: four round sardinellas (*Sardinella aurita*), two wild European seabass, one Mediterranean horse mackerel (*Trachurus mediterraneus*), one blotched picarel (*Spicara maena*) and one pompano (*Trachinotus ovatus*). Lastly, the only positive fish from the extractive fisheries group was a bogue (*Boops boops*). Mean total body length for each species in which positive individuals had been detected indicates that the wild individuals analyzed were adults, the vast majority of them having reached sexual maturity. In the case of farmed species, although they were not sexually mature individuals in all cases, they could be considered young adults, with a minimum on-growing period in sea pens of approximately 20 months (Table 2). Prevalence for each host species is also recorded in Table 2.

### 3.2. Molecular Identification at the 18S rRNA Gene

Sequence and phylogenetic analysis at the 18S rRNA gene identified two species of *Cryptosporidium*, *C. molnari* (76.5%; 13/17) and *C. ubiquitum* (11.8%; 2/17), one isolate similar to *C. scophthalmi* (5.9%; 1/17) and one unidentified *Cryptosporidium* (5.9%; 1/17).

**Figure 2:**
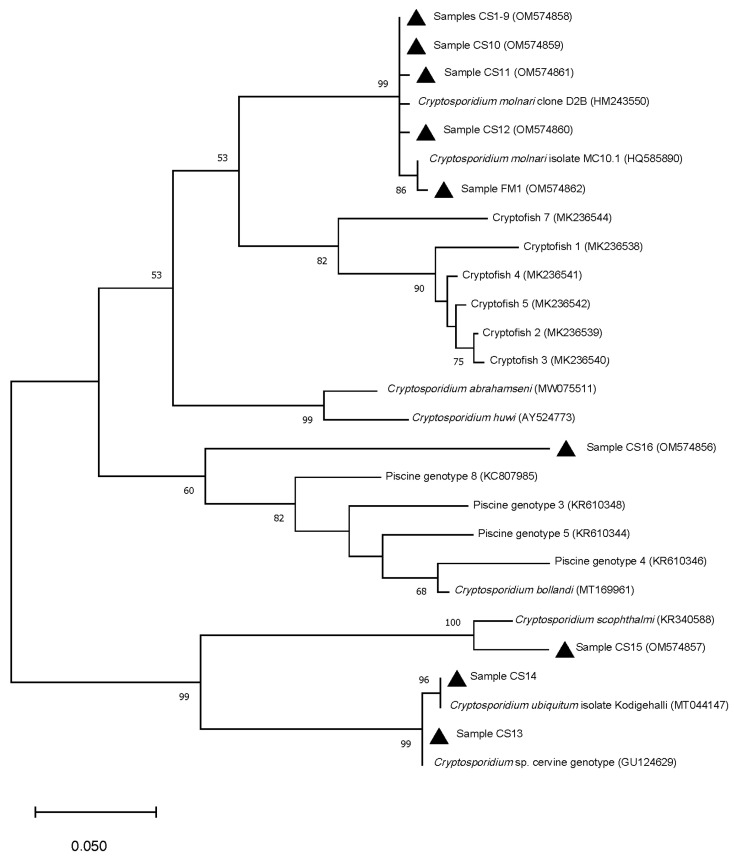
Phylogenetic relationships between *Cryptosporidium* isolates from this study (▲) and other *Cryptosporidium* species and genotypes inferred by Maximum-Likelihood (ML) method of 18S rRNA gene sequences (277 bp). Percentage support (>50%) from 1000 replicates (bootstrap test) is indicated at the left of the supported node. Scale bar refers to a phylogenetic distance of 0.05 nucleotide substitutions per site.

*C. molnari* was identified in the three groups studied and in seven different host species (Table 2). Nine out of 13 positive individuals identified as *C. molnari* (samples CS1–9) were 100% identical and exhibited a genetic similarity of 99.80% with the sequence deposited in the GenBank under the accession number HM243550. This sequence diverged from isolates CS1–9 in a unique single nucleotide variant (SNV). Samples CS10–12 exhibited a genetic similarity between 99.50–99.61% with the same reference sequence and presented the same SNV together with others. Finally, one *C. molnari* isolated from an extractive fisheries specimen (sample FM1) was genetically closer to the sequence deposited in the GenBank under the accession number HQ585890 (Table 3, Figure 2).

Two sequences were identified as zoonotic *C. ubiquitum*, both in European seabass. One of them (sample CS14) presented 100% genetic similarity with the sequence deposited in GenBank under accession number MT044147. The other sample (CS13) exhibited 100% genetic similarity with a sequence from *Cryptosporidium* cervine genotype (GU124629), the old name for *C. ubiquitum* (Table 3, Figure 2). Attempts at amplifying these samples at the 60-kDa glycoprotein gene (GP60; [31]) failed and isolates could not be sub-typed.

One sequence from a cultivated meagre (sample CS15) showed 97.21% genetic similarity with *C. scophthalmi* reference sequence (KR340588). Phylogenetic analysis revealed a genetic distance of 4.8% (Table 3, Figure 2).

Finally, sample CS16 from one round sardinella was closer to *C. bollandi*, although it only presented 88.16% genetic similarity with this species reference sequence (MT169961). Genetic distance between sample CS16 and *C. bollandi* was 25.3% and phylogenetic analysis inferred a new clade for this sample, highly divergent from all the species and genotypes previously reported (Table 3, Figure 2).

### 3.3. Molecular Identification at the Actin Gene

**Figure 3:**
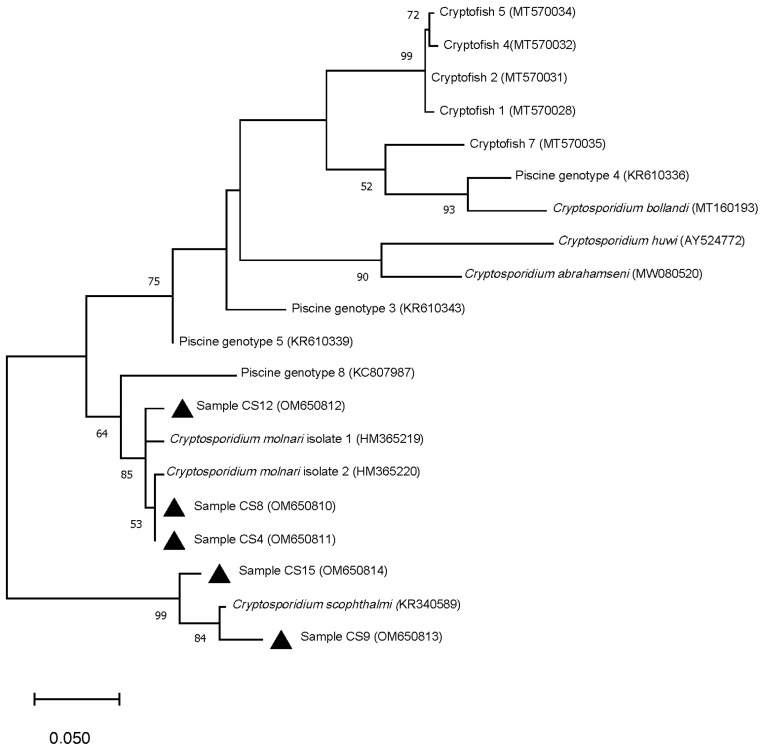
Phylogenetic relationships between *Cryptosporidium* isolates from this study (▲) and other *Cryptosporidium* species and genotypes from fish host inferred by Maximum-Likelihood (ML) method of actin gene sequences (192 bp). Percentage support (>50%) from 1000 replicates (bootstrap test) is indicated at the left of the supported node. Scale bar refers to a phylogenetic distance of 0.05 nucleotide substitutions per site.

From 17 positive isolates, five were successfully amplified and sequenced at the actin gene locus. For the following four samples, there was concordance with the 18S rRNA gene identification: samples CS4, CS8 and CS12 corresponded to *C. molnari*, while sample CS15 presented a nucleotide sequence more closely related to *C. scophthalmi* (genetic distance of 3.8%), as occurred at the 18S rRNA gene locus, although in this case the isolate clustered separately (Figure 3). Isolate CS9, which was typed as *C. molnari* at the 18S rRNA gene, was more closely related to *C. scophthalmi* at the actin gene (genetic distance of 2.7%), suggesting a probable mixed infection in this specimen (Table 3, Figure 3).

## 4. Discussion

The *Cryptosporidium* prevalence measured in adult wild fishes (3.9 ± 1.2%) can be considered low, with comparable results to those reported previously in Papua New Guinea (1.45% [26]), Australia (2.4% [32]) and in different European seas (2.3–3.2% [33]). The study of wild fish allowed us to check not only commercial fishes from markets but also synanthropic fishes living in the vicinity of aquaculture farms. The inclusion of the synanthropic group could explain the slightly higher prevalence in the present study, due to higher population densities in the surroundings of aquaculture facilities, attracted by the abundance of food [34,35]. This fact could also explain the difference in prevalence detected between the synanthropic and extractive fishery groups (6.1% and 0.9%, respectively).

The *Cryptosporidium* spp. prevalence in the cultivated group was 4.8 ± 1.8%, corresponding to *C. molnari* infections in young adults of gilthead seabream and European sea bass, and a *C. scophthalmi*-like isolate from a meagre. The prevalence obtained in gilthead sea bream (5.12%) is comparable to the previously detected prevalence in individuals of similar weights in offshore on-growing systems [36], and lower than the prevalence detected in younger fish, which are considered highly susceptible, as is generally observed in human and animal cryptosporidiosis [5,37]. Prevalence in European sea bass has been studied mainly among fingerlings and juveniles, with variable prevalence according to fish age and weight [10,36]. Available data in older individuals reported 0% prevalence [33,36]. In the present study, only young adults from offshore on-growing farms have been sampled and analyzed, showing a 3.57% prevalence.

The low prevalence in this survey could have been explained by the age of the fishes. A higher prevalence could be expected in younger individuals. However, fishes close to commercial size are more relevant for public health. Future studies should consider the possibility of sampling throughout the year, to determine whether seasonality may influence prevalence. Most positive fishes in this study corresponded to *C. molnari* isolates (76.5%), contrasting with previous surveys in marine fish, where different piscine genotypes and zoonotic *C. parvum* were more frequent [26,32,33]. This fact could be explained by the fact that sampling comprised cultivated gilthead sea bream and European sea bass (type hosts for *C. molnari*), which were environmentally related to the synanthropic populations, facilitating the transmission of this parasite, as occurs in other parasitic infections (e.g., sea louse in Atlantic Salmon, [38,39]). Therefore, *C. molnari* was detected in both cultivated species, but also in wild synanthropic round sardinella, blotched picarel, pompano and Mediterranean horse mackerel, expanding the range of known hosts for *C. molnari*. Nine out of thirteen *C. molnari* isolates were homologues at 18S rRNA partial sequences obtained and differed in one SNV from other *C. molnari* available sequences. From those, seven samples corresponded to cultivated and synanthropic individuals from the same location (Farm 4, see Table 3); another two corresponded to wild synanthropic individuals captured in other locations (Farms 1 and 3), one Mediterranean horse mackerel and one round sardinella, both pelagic migratory species, commonly associated with marine farms [34]. It is well known that coastal aquaculture facilities attract wild fish populations that forage on waste fish feed [35]. Movement of these farm-aggregating populations acts upon connecting farms and other marine areas [35,40,41]. This behavior could potentially enable the transmission of pathogens between farms and to wild populations through farm-aggregating wild fish movements [35]. Molecular data at the 18S rRNA gene from this study highlights the possibility of the spreading of *Cryptosporidium* spp. between different locations by migratory species that inhabit the surroundings of aquaculture facilities. Sequences at the actin gene for *C. molnari* isolates were in concordance with 18S rRNA data, except for a synanthropic round sardinella, which seemed to present a mixed infection with *C. molnari* (18S rRNA gene) and other isolates more similar to *C. scophthalmi* (actin gene).

No previous data exist for *Cryptosporidium* prevalence or species in meagre. In this study, only one individual out of 25 was found to be infected by one *Cryptosporidium* similar to *C. scophthalmi*. *C. scophthalmi* was originally described in cultured turbot [42] by microscopic examination and histological techniques. To date, the unique molecular data for this species has been reported by Costa and Saraiva (2015) [12] in the same host. Isolate from meagre in this study exhibited a genetic distance of 4.8% (18S rRNA gene) and 3.8% (actin gene) with the turbot’s sequences. It would be necessary to conduct a targeted study on meagres to better characterize this *C. scophthalmi*-like isolate.

*C. ubiquitum* was detected in two European sea bass. One specimen belonged to the cultivated group while the other was part of the synanthropic group, both coming from the same location (Farm 2). It is important to highlight that the escape of fish from marine aquaculture farms has been reported around the world and in different cultivated species, including the European sea bass [43,44]. Therefore, we cannot exclude the possibility that synanthropic individuals of European sea bass in this study were cultured individuals that had escaped from farms. To our knowledge, this is the first time that *C. ubiquitum* has been detected in a fish host. *C. ubiquitum*, formerly known as *Cryptosporidium* cervine genotype, is a widespread zoonotic emergent species, able to affect a wide range of hosts, greater than that of other *Cryptosporidium* species (domestic and wild ruminants, rodents, carnivores and primates [45]). In Spain, *C. ubiquitum* reports are scarce, both in animals and humans. It has been reported in lambs and in an adult sheep [46,47], in a red fox [48] and in a 6-year-old child [49]. Moreover, its presence in water sources has also been reported. In Spain, *C. ubiquitum* has been detected from an influent of a wastewater treatment plant [50]. The presence of *C. ubiquitum* in water sources potentially explains its presence in fish hosts, as seems to occur with *C. parvum* [33]. Zoonotic *Cryptosporidium* spp. can reach the marine environment from runoff or sewage water, as they resist the disinfectants commonly used in the water industry [50]. In this study, the sequences at the 18S rRNA gene of the two positive isolates were 100% identical to two sequences from domestic cattle from other countries. However, attempts to sub-type these isolates failed, making it difficult to identify a potential origin. The findings of the present survey imply a new concern for public health, of special importance in Spain, the third highest fish consuming country within the European Union [23]. Histopathological analysis would have been necessary to determine whether this was a possible natural infection or, on the contrary, the fish were only acting as mechanical transporters [21]. However, the mere presence of *C. ubiquitum* in fish gastrointestinal tracts may pose a risk of transmission to humans. Although European sea bass is not commonly consumed undercooked, it still remains a risk while handling [51]. Moreover, other fish species, which are commonly consumed whole, raw or undercooked, could potentially harbor this zoonotic species.

Finally, sample C16 from a wild synanthropic round sardinella was identified as a *Cryptosporidium* sp. highly divergent from known species/genotypes. As inferred by the ML method at the 18S rRNA gene (Figure 2), it seems to constitute a new clade. Unfortunately, attempts to amplify the actin locus failed for this isolate. Although molecular data at the 18S rRNA gene could be indicative of a new *Cryptosporidium* species, it would be necessary to detect more isolates and to perform more molecular and morphological studies.

## 5. Conclusions

This study provides new data on the molecular characterization of *Cryptosporidium* spp. in marine fish, identifying new host species for *C. molnari* and evidencing the high diversity of this parasite in fish. Two potential new genotypes/species have been detected, although further studies are necessary to characterize them. Finally, the detection of zoonotic *C. ubiquitum* in fish could represent a new source of food-borne cryptosporidiosis, highlighting the importance of these studies in risk assessment for fish consumption.

## Figures and Tables

**Table 1:** Marine fish collected in the present study depending on the species, group and location.

Cultivated Fish						
Scientific Name	Common Name	Farm 1	Farm 2	Farm 3	Farm 4	Total
*Argyrosomus regius*	Meagre	-	13	11	-	24
*Dicentrarchus labrax*	European seabass	24	26	19	15	84
*Sparus aurata*	Gilthead seabream	9	-	-	30	39
Total		33	39	30	45	147
Synanthropic fish						
Scientific name	Common name	Farm 1	Farm 2	Farm 3	Farm 4	Total
*Belone belone*	Garfish	3	-	-	-	3
*Boops boops*	Bogue	1	-	1	4	6
*Chelon labrosus*	Thicklip grey mullet	-	-	1	-	1
*Dicentrarchus labrax*	European seabass	-	1	-	9	10
*Diplodus puntazzo*	Sharpsnout seabream	-	-	1	-	1
*Diplodus sargus*	White seabream	-	4	-	-	4
*Diplodus vulgaris*	Common two-banded seabream	-	5	9	2	16
*Mullus barbatus*	Red mullet	-	-	1	-	1
*Pagellus acarne*	Axillary seabream	-	17	8	-	25
*Pagellus erythrinus*	Common pandora	-	1	2	1	4
*Sardinella aurita*	Round sardinella	13	-	3	9	25
*Scomber japonicus*	Chub mackerel	-	1	2	-	3
*Serranus cabrilla*	Comber	-	-	-	1	1
*Sparus aurata*	Gilthead seabream	-	2	-	-	2
*Spicara maena*	Blotched picarel	3	-	-	8	11
*Spondyliosoma cantharus*	Black seabream	-	-	-	1	1
*Trachinotus ovatus*	Pompano	1	-	1	-	2
*Trachurus mediterraneus*	Mediterranean horse mackerel	8	11	6	6	31
Total		29	42	35	41	147
Fish from extractive fisheries						
Scientific name	Common name	Market 1	Market 2	Market 3	Market 4	Total
*Argentina sphyraena*	Argentine	-	-	-	1	1
*Argyrosomus regius*	Meagre	-	-	-	1	1
*Arnoglossus imperialis*	Imperial scaldfish	-	-	-	1	1
*Boops boops*	Bogue	-	3	-	-	3
*Chelidonichthys cuculus*	Red gurnard	-	-	1	-	1
*Citharus linguatula*	Spotted flounder	-	-	3	1	4
*Conger conger*	European conger	-	-	-	2	2
*Diplodus annularis*	Annular seabream	-	2	-	-	2
*Diplodus vulgaris*	Common two-banded seabream	-	-	-	1	1
*Helicolenus dactylopterus*	Blackbelly rosefish	-	-	-	1	1
*Labrus merula*	Brown wrasse	-	-	-	1	1
*Lepidotrigla cavillone*	Large-scaled gurnard	-	-	-	1	1
*Lophius budegassa*	Blackbellied angler	-	-	1	1	2
*Merluccius merluccius*	European hake	-	2	5	4	11
*Micromesistius poutassou*	Blue whiting	-	-	2	3	5
*Mullus barbatus*	Red mullet	-	1	6	2	9
*Mullus surmuletus*	Surmullet	-	-	1	2	3
*Pagellus acarne*	Axillary seabream	-	1	-	-	1
*Pagellus erythrinus*	Common pandora	-	3	1	1	5
*Peristedion cataphractum*	African armoured searobin	-	-	-	1	1
*Phycis blennoides*	Greater forkbeard	-	-	4	4	8
*Phycis phycis*	Forkbeard	-	-	-	1	1
*Sarpa salpa*	Salema	-	-	-	1	1
*Scomber scombrus*	Atlantic mackerel	19	1	-	2	22
*Scyliorhinus canicula*	Lesser spotted dogfish	-	-	3	-	3
*Serranus cabrilla*	Comber	-	-	-	1	1
*Serranus hepatus*	Brown comber	-	-	1	1	2
*Trachinus draco*	Greater weever	-	-	1	-	1
*Trachurus mediterraneus*	Mediterranean horse mackerel	-	2	-	1	3
*Trisopterus luscus*	Pouting	-	2	7	2	11
*Uranoscopus scaber*	Stargazer	-	-	-	1	1
Total		19	17	36	38	110

The symbol “-“ means that no specimens of that fish species were sampled in the corresponding farm or fish market.

**Table 2:** Prevalence of *Cryptosporidium* spp. and *C. molnari* for each species in which positive individuals were detected. Study group, mean weight, total body length and reference total body length at sexual maturity are indicated.

Host Species	N	Mean Weight ± STD (g)	Mean Total Body Length ± STD (cm)	Mean Total Body length at Sexual Maturity (cm) ^a^	*Cryptosporidium* Prevalence (%)	*C. molnari* Prevalence (%)	Group
*Dicentrarchus labrax* (European seabass)	84	384.51 ± 186.86	31.15 ± 5.52	36.1	4.76 (4/84)	3.57 (3/84)	C
	10	318.74 ± 85.83	33.36 ± 1.94		20 (2/10)	10 (1/10)	S
*Sparus aurata* (Gilthead seabream)	39	279.25 ± 75.58	24.45 ± 2.13	36.5	5.12 (2/39)	5.12 (2/39)	C
	2	509.57 ± 312.47	31.6 ± 7.64		0	0	S
*Argyrosomus regius* (Meagre)	24	474.68 ± 269.92	41.98 ± 11.99	61.6 [29]	4.17 (1/24)	0	C
*Sardinella aurita* (Round sardinella)	25	96.84 ± 32.45	22.28 ± 2.47	18.8	16 (4/25)	12 (3/25)	S
*Boops boops* (Bogue)	6	105.26 ± 40.79	21.5 ± 3.89	14.3	0	0	S
	3	76.56 ± 24.31	19.77 ± 2.42		33.34 (1/3)	33.34 (1/3)	EF
*Spicara maena* (Blotched picarel)	11	77.05 ± 38.67	18.85 ± 3.77	11.5	9.09 (1/11)	9.09 (1/11)	S
*Trachinotus ovatus* (Pompano)	2	139.64 ± 53.97	24.95 ± 1.48	30 [30]	50 (1/2)	50 (1/2)	S
*Trachurus mediterraneus* (Mediterranean horse mackerel)	31	164.28 ± 63.57	26.47 ± 3.82	20	3.23 (1/31)	3.23 (1/31)	S
	3	65.65 ± 21.67	19.5 ± 2.18		0	0	EF

^a^ Data extracted from: https://www.fishbase.se/search.php (accessed on 13 April 2022). C: cultivated fish; EF: fish from extractive fisheries; N: total number of specimens analysed for each host species and group; S: synanthropic fish; STD: standard deviation.

**Table 3:** *Cryptosporidium* spp. identified in marine fish at the 18S rRNA and actin genes.

Sample	Host Species	Group	Farm/ Market	18S rRNA			Actin		
				Identification	Most Similar Sequence	% Identity/SNVs	Identification	Most Similar Sequence	% Identity/SNVs
CS1	*Dicentrarchus labrax*	C	4	*C. molnari*	HM243550	99.80/1	---	---	---
CS2	*Dicentrarchus labrax*	C	4	*C. molnari*	HM243550	99.80/1	---	---	---
CS3	*Dicentrarchus labrax*	C	4	*C. molnari*	HM243550	99.80/1	---	---	---
CS4	*Sparus aurata*	C	4	*C. molnari*	HM243550	99.80/1	*C. molnari*	HM365220	99.25/2
CS5	*Spicara maena*	S	4	*C. molnari*	HM243550	99.80/1	---	---	---
CS6	*Sardinella aurita*	S	4	*C. molnari*	HM243550	99.80/1	---	---	---
CS7	*Dicentrarchus labrax*	S	4	*C. molnari*	HM243550	99.80/1	---	---	---
CS8	*Trachurus mediterraneus*	S	1	*C. molnari*	HM243550	99.80/1	*C. molnari*	HM365220	98.83/3
CS9	*Sardinella aurita*	S	3	*C. molnari*	HM243550	99.80/1	*C. scophthalmi*-like	KR340589	95.77/33 ^a^
CS10	*Sparus aurata*	C	1	*C. molnari*	HM243550	99.50/3	---	---	---
CS11	*Sardinella aurita*	S	3	*C. molnari*	HM243550	99.60/2	---	---	---
CS12	*Trachinotus ovatus*	S	3	*C. molnari*	HM243550	99.61/2	*C. molnari*	HM365220	98.20/11
FM1	*Boops boops*	EF	2	*C. molnari*	HQ585890	99.80/1	---	---	---
CS13	*Dicentrarchus labrax*	S	2	*C. ubiquitum*	GU124629	100	---	---	---
CS14	*Dicentrarchus labrax*	C	2	*C. ubiquitum*	MT044147	100	---	---	---
CS15	*Argyrosomus regius*	C	2	*C. scophthalmi*-like	KR340588	97.21/14	*C. scophthalmi*-like	KR340589	96.11/21 ^b^
CS16	*Sardinella aurita*	S	4	Unidentified *Cryptosporidium*	MT169961	88.16/56	---	---	---

^a^ Query cover: 95%. ^b^ Query cover: 92%. C: cultivated fish; EF: fish from extractive fisheries; S: synanthropic fish; SNV: single nucleotide variant. Symbol “---” means that actin gene couldn’t be amplified for that sample.

## Data Availability

The datasets analyzed during the current study are available from the corresponding author on reasonable request.
